# Contact Photolithography at Sub-Micrometer Scale Using a Soft Photomask

**DOI:** 10.3390/mi10080547

**Published:** 2019-08-18

**Authors:** Chun-Ying Wu, Heng Hsieh, Yung-Chun Lee

**Affiliations:** Department of Mechanical Engineering, National Cheng Kung University, Tainan 70101, Taiwan

**Keywords:** soft photomask, contact photolithography, black photoresist, ultraviolet patterning, sub-micrometer scale

## Abstract

This paper proposes a method for improving the patterning resolution of conventional contact photolithography from the micrometer, down to the sub-micrometer scale. The key element is a soft polydimethylsiloxane (PDMS) photomask, which is first replicated from a silicon mold and then patterned with a black photoresist (PR) layer to selectively block ultraviolet (UV) light. This soft PDMS photomask can easily form an intimate and conformable contact with a PR-coated substrate and hence can perform contact photolithography with high pattern resolution. The fabrication processes of this black-PR/PDMS soft photomask are experimentally carried out. Using the fabricated soft photomask, UV patterning by contact photolithography with the smallest line-width of 170 nm over a 4” wafer area was successfully achieved. The advantages and potentials of this new type of contact photolithography will be addressed.

## 1. Introduction

Photolithography technologies have been successfully developed in the past few decades and are now the most mature and dominant method for micro- and nanofabrication in the industry [1,2,3,4]. There are basically two categories of photolithography. The image-projection photolithography utilizes an ultraviolet (UV) light source and an optical image projection system to project a de-magnified pattern defined by a glass or quartz photomask onto a photo-sensitive layer (known as a photoresist (PR) layer) deposited on a substrate. After developing, the patterned PR microstructures are formed and the subsequent material processes can be followed. One of the most important advantages of projection photolithography is its high resolution and a line-width down to a few tens of nanometer can be achieved nowadays in the semiconductor industry [5,6]. However, both capital investments and running costs for projection photolithography are very high, which limits its availability and makes it less accessible to general users.

The second type of photolithography, known as contact or proximity photolithography, brings a photomask either in contact or in close proximity to a PR layer so that the UV light passing through the photomask can directly form a 1:1 image on the PR layer. Contact or proximity photolithography systems are relatively inexpensive and the UV patterning can be carried out over a large area with a high throughput [7]. However, the resolution for contact or proximity photolithography is quite limited and the typical line-width is above 1 μm or so, which limits its applications [8]. This limitation is because, to achieve a patterning resolution at sub-micrometer or even nanometer scale, one needs to form an intimate contact between the photomask and the PR-coated substrate and to prevent any in-between gap that might cause light diffraction [9]. This is practically impossible, considering both photomasks and substrates are typically made of hard materials. Maintaining their surface flatness at a sub-micrometer or nanometer scale over wafer-size areas is extremely difficult, if not impossible. Furthermore, preparing a photomask with high pattern resolution can be technically challenging and financially expensive. Not to mention the lifetime of photomasks will be greatly reduced due to constant rubbing with substrates or contamination by PR in contact photolithography.

**Figure 1:**
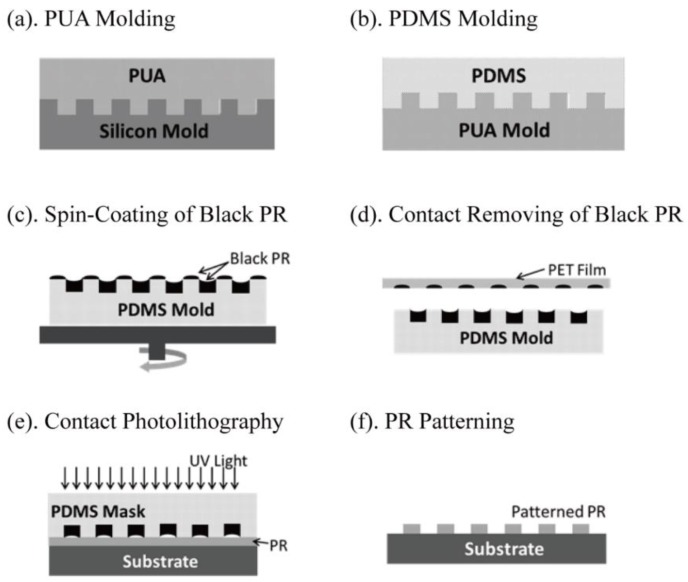
Flow charts for preparing a black-photoresist embedded soft photomask to carry out high-resolution contact lithography.

The goal of this paper is to solve the above-mentioned problems and to bring the patterning resolution of contact photolithography down to the sub-micrometer scale. It is based on a new type of black-photoresist embedded soft photomask, proposed early by this group [10] for patterning micro-scaled PR structures on non-flat sapphire substrates. Figure 1 shows schematically the processes of making such a soft photomask. First of all, a silicon mold is prepared using standard photolithography. Wafer-size (6”, 8”, or even 12”) silicon molds with sub-micrometer and nanometer scaled surface features can be realized from the semiconductor industry. As shown in Figure 1a,b, a polyurethane acrylate (PUA) mold is first replicated from the silicon mold and a polydimethylsiloxane (PDMS) mold is then subsequently replicated from the PUA mold. The PDMS mold will then have the same surface features as the silicon master mold. A black photoresist, which is basically UV-curable solvent, containing dispersive carbon-black nanoparticles, is spin-coated on the PDMS mold’s surface. After curing, the solidified black PR can fill the concave cavities on the PDMS surface, as shown in Figure 1c. Some black PR may remain sitting on the top surface of the PDMS mold but can be removed by a polyethylene terephthalate (PET) film through a contact adhesion process, as shown in Figure 1d. Finally, the PDMS mold containing the black PR in its concave surface area can be used as a soft photomask in contact photolithography to obtain patterned PR structures, as shown in Figure 1e,f.

There are many important advantages in using this soft photomask for contact photolithography. First of all, the soft PDMS photomask can easily form an intimate and conformal contact with a substrate, even when the substrate surface is not perfectly flat. This is critical for achieving high-resolution patterning in real industrial applications. Secondly, since soft PDMS photomasks can be repeatedly fabricated from one single silicon mold using materials and equipment that are readily available, the cost is drastically reduced. Finally, using a large-area silicon mold, one can achieve large-area patterning of PR structures with small line-width and high throughput. In short, this soft photomask approach preserves all of the important advantages in contact photolithography and, in the meantime, overcomes the major challenges and difficulties encountered before. In this work, experiments are carried out to explore the capability of sub-micrometer patterning using the black-PR/PDMS soft photomasks, as well as their potential for future industrial applications.

## 2. Experiments and Results

**Figure 2:**
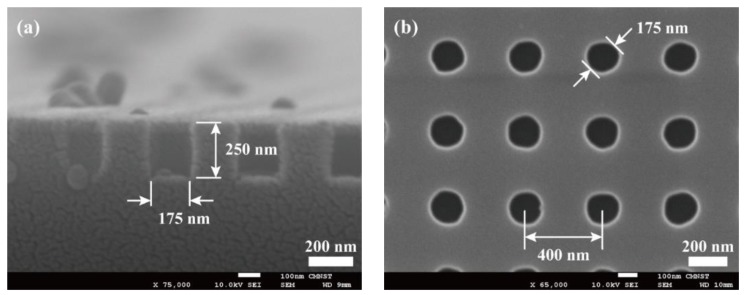
Scanning electron microscope (SEM) images of a silicon mold with squarely arrayed micro-holes which are 175 nm in diameter, 250 nm in depth, and 400 nm in center-to-center pitch; (**a**) cross-sectional and (**b**) top views.

**Figure 3:**
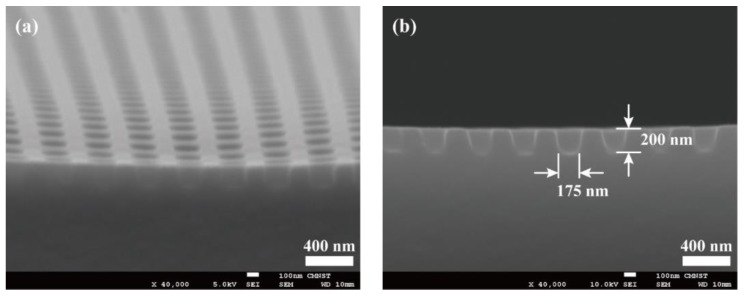
Images of a (**a**) tilted and (**b**) cross-sectional view of the hard polydimethylsiloxane (hPDMS)/PDMS mold replicated from a polyurethane acrylate (PUA) mold. The arrayed holes on mold’s surface have a diameter of 175 nm and a depth of 200 nm.

To investigate the capabilities of sub-µm scale UV patterning using the black-PR/PDMS soft molds, an 8” silicon mold was first fabricated by a local semiconductor foundry. The silicon mold surface contained squarely arrayed holes, which are 175 nm in diameter, 250 nm in depth, and 400 nm in center-to-center pitch. Figure 2a,b are the scanning electron microscope (SEM) image of these hole-arrayed surface structures. Following the standard molding processes, a PUA (MINS-301RM, Minuta Technology Co., Ltd., Seoul, Korea) mold was negatively replicated from the silicon mold. PUA is known for its excellent molding capability at the sub-micrometer or even nanometer scale [11,12,13]. However, its high elastic modulus and hardness disqualify it as a soft photomask for contact photolithography. Using the PUA mold, we would like to obtain a PDMS mold, which has high deformability. However, to faithfully transfer the sub-micrometer features (around 100 nm), a hard PDMS or h-PDMS (VDT-731, SIP6831.1, SIT-7900, HMS-301, Gelest, Inc., Morrisville, PA, USA) [14,15,16] was first spin-coated and then cured on top of the PUA mold. It was then followed by the regular molding procedures of PDMS (Sylgard^®^ 184, Dow Corning, Midland, MI, USA) to complete the h-PDMS/PDMS mold [17,18,19]. SEM images of the PDMS mold are displayed in Figure 3. As can be seen in Figure 3, the arrayed holes replicated on the PDMS mold surface had a diameter of 175 nm and a depth of 200 nm, which were slightly different from their counterparts in the silicon mold and could be recognized as a certain degree of dimensional change due to the molding processes and materials’ shrinkage.

**Figure 4:**
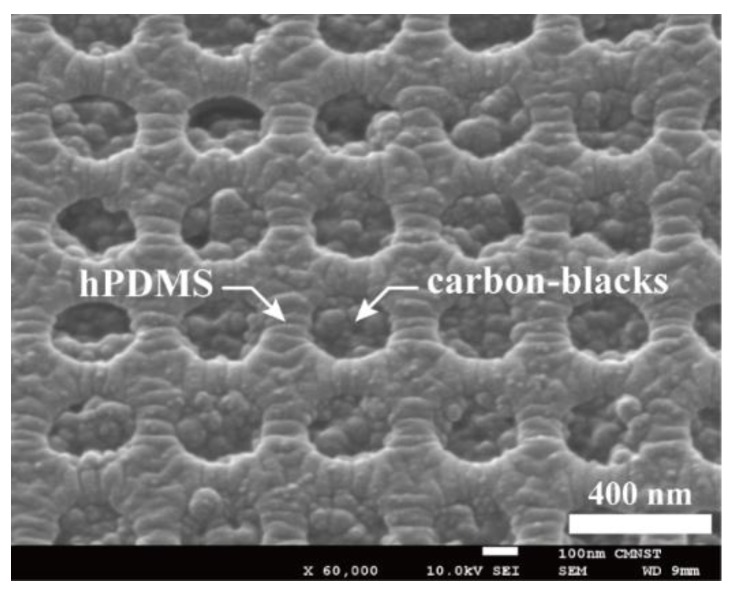
Spin-coating of black photo-resist and contact removing by a polyethylene terephthalate (PET) film, the holes on the surface of the hPMDS/PDMS mold are now filled with carbon-blacks to block UV light transmission.

To convert this PDMS mold into a soft photo-mask, a black photoresist needs to be spin-coated on the PDMS mold. To improve surface adhesion, the PDMS mold was first treated with oxygen (O_2_) plasma at a power of 30 watts for 5 min. After that, black-photoresist EK520 (Everlight Chemical Industrial Corporation, Taipei, Taiwan), which contained 22.5% of carbon black in the weight ratio, was spin-coated on the PDMS mold surface. We adjusted the spinning speed so that the micro-holes on the PDMS mold’s surface could be filled with black PR, and on the other hand, the black PR, which may remain sitting on the top surface of PDMS mold could be minimized. This spinning speed depended on parameters such as the black-PR’s viscosity, the surface conditions of the PDMS mold, and the geometrical dimensions of the holes. A spin-coating speed of 7000 rpm was used, based on trial and error. The black-PR coated PDMS mold was then thermally cured by a hot plate at a temperature of 70 °C for 15 min. After solidification, the black PR filling inside the holes would shrink a little bit and hence further withdraw away from the top surface of PDMS mold. To remove the black PR remaining on PDMS mold’s top surface, a 175 µm thick PET film was brought into contact with the PDMS mold. A pressure of 0.2 MPa was applied to the PET/PDMS interface at a temperature of 75 °C for 2 min. The black PR sandwiched in between the PET film and the PDMS’s top surface was then adhered to the PET film and could be removed after peeling off the PET film. A black-PR embedded PDMS soft mold was now completed. Figure 4 shows the SEM images of the soft mold surface and solidified carbon-black particles can be observed inside the holes.

Before conducting contact photolithography, the optical characteristics of the black PR, acting as a UV-blocking layer in the soft photomask, are examined first. The EK520 black PR was spin-coated on a 0.7 mm thick quartz plate. After the same curing process given before, the optical transmission coefficient of the black-PR coated quartz plate was measured and then divided by the transmission coefficient of the quartz plate without the black PR. The transmission coefficients for a 320 nm thick EK520 black PR film was experimentally determined to be 1.48 % at a light wavelength of 365 nm (I-line). Based on Beer–Lambert law of light absorption in materials, the optical density (OD) for a cured EK520 black PR layer with a thickness of 1 µm was around 5.7. For a 200 nm thick EK520 black PR layer used in the black-PR/PDMS mold we prepared, the transmission coefficient was estimated at around 7.2%. This was the contrast we could have when using this black-PR/PDMS photomask for contact photolithography. The characteristics of the photoresist layer and UV dosage used for UV patterning were chosen accordingly.

**Figure 5:**
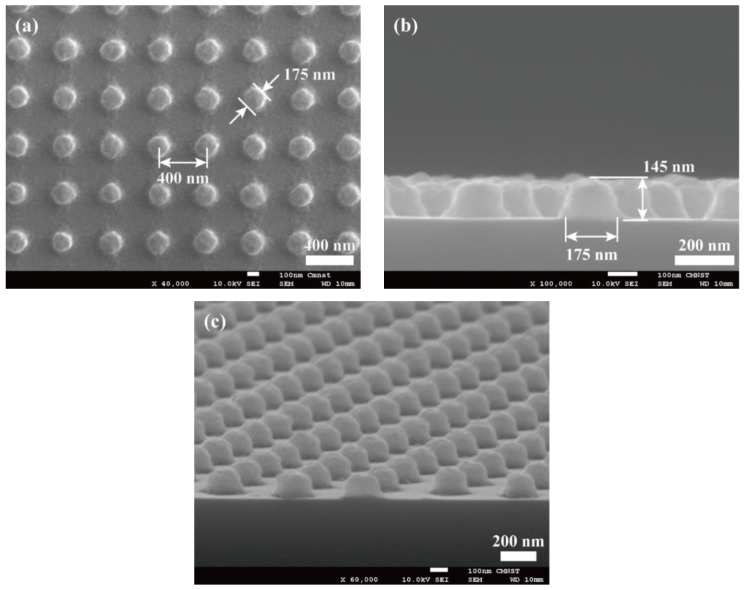
Images of the patterned photoresist (PR) micro-structures by contact lithography using the black-photoresist embedded soft photomask; (**a**) top, (**b**) cross-sectional, and (**c**) tilted views.

To test the patterning capability of the black-PR/PDMS photomask at the sub-micrometer scale, a positive-tone photoresist AZ GXR 601 (AZ Electronic Materials Co., Ltd., Taipei, Taiwan) was used. The AZ GXR 601 PR was first diluted with propylene glycol monomethyl ether acetate (PGMEA, AZ Electronic Materials Co., Ltd., Taipei, Taiwan) at a weight ratio of 1:3 and then spin-coated on a 4” silicon wafer at a spinning speed of 3000 rpm. The PR film thickness was measured and was 150 nm. After soft baking for 1 min at a temperature of 100 °C, the PR coated silicon substrate was brought in contact with the black-PR/PDMS photomask. A vacuum chamber and a mechanical mechanism were specifically designed and constructed to ensure the soft photomask was in good contact with the silicon substrate. A collimated 6” UV light source (ELS-201SA, ELS System Technology Co., Zhubei, Taiwan) with a wavelength of 365 nm was incident through the black-PR/PDMS photomask to expose the AZ GXR 601 PR layer at a power intensity of 30 mW/cm^2^ for 0.3 s. After developing in AZ 300 MIF developer (AZ Electronic Materials Co., Ltd., Taipei, Taiwan) for 10 s and then hard baking at 120 °C for 2 min, PR microstructures were obtained on the silicon substrate. SEM images of the obtained PR microstructures are shown in Figure 5. The obtained PR microstructures were arrayed micro-pillars with a diameter around 175 nm, a height of 145 nm, and a center-to-center pitch of 400 nm. This demonstrated the capability of the black-PR/PDMS photomask for patterning micro-structures at the sub-micrometer scale.

## 3. Conclusions

In this work, we demonstrated how to bring the patterning resolution of contact photolithography from the current status of 1 µm, down to the order of 100 nm. The key factor was to replace conventional metal-coated glass/quartz photomasks by a soft PDMS mold embedded with a patterned black-photoresist layer. This soft PDMS photomask can be fabricated in the laboratory using a silicon master mold and a series of processing methods, including molding and contact pattern transfer. The deformable and compliant nature of this soft PDMS photomask ensures its intimate and conformable contact with a PR-coated substrate and allows parallel UV light to directly transfer the photomask’s pattern to the PR layer. UV patterning on a 150 nm thick positive-tone photoresist layer with a characteristic feature size of 175 nm over a 4” silicon wafer was successfully achieved using simple equipment readily available in laboratories and the industry.

Since the PDMS photomask was replicated from a silicon master mold, the smallest line-width and the largest patterning area one can achieve using this new type of contact photolithography were mainly determined by the fabrication capabilities of the silicon molds. In the semiconductor industry, 8” and 12” silicon wafers with a smallest line-width ranging from 1 µm to few tens of nm are quite common. However, to block UV light, the black photoresist layer has to have a certain thickness in order to obtain the optical contrast needed for UV patterning. This means a higher aspect ratio in the silicon mold’s surface microstructures is needed when applying this method for smaller line-width patterns. Research on further reducing the patterning resolution to below 100 nm is under study.
